# Comparative Evaluation of a Domestic Automatic Milking System and a Commercial System: Effects of Parity on Milk Performance and System Capacity

**DOI:** 10.3390/ani15243649

**Published:** 2025-12-18

**Authors:** Dong-Hyun Lim, Jun Sik Eom, Seong Min Park, Jihoo Park, Dong Hyeon Kim, Taejeong Choi, Young Kyung Choi, Jongseon Kim, Younghoon Kim

**Affiliations:** 1 Dairy Science Division, National Institute of Animal Science, Rural Development Administration, Cheonan-si 31000, Republic of Korea; idh1974@korea.kr (D.-H.L.); eomjs0606@korea.kr (J.S.E.); bek9love@korea.kr (S.M.P.); jihoopk@korea.kr (J.P.); kimdh3465@korea.kr (D.H.K.); choi6695@korea.kr (T.C.); 2 Dawoon Co., Ltd., 22, Bodojin-ro 42beon-gil, Seo-gu, Incheon 22847, Republic of Korea; dawoonel@naver.com (Y.K.C.); jongseonk@dawoon.com (J.K.); 3 Department of Agriculture Biotechnology, Research Institute of Agriculture and Life Science, Seoul National University, Seoul 08826, Republic of Korea

**Keywords:** automatic milking system, milking performance, parity, dairy cows

## Abstract

This study evaluated milking characteristics and milk productivity of a domestically developed automatic milking system (AMS-K) and compared its performance with a commercially imported AMS (AMS-C), under identical farm management conditions. Milking performance of AMS-K was monitored over a three-month operating period, and a comparative analysis was subsequently conducted using a total of 50 Holstein cows, with 25 cows allocated to each system based on similar parity, days in milk, and milk yield. During the three-month operation of AMS-K, milk yield per milking significantly increased in from 13.81 kg in the 1st month to 15.99 kg in the 3rd month, and daily milk yield increased by 4.01–7.52% compared with the initial operating period. Milking frequency decreased from 2.53 to 2.27 times per day, but remained higher than conventional twice-daily milking. Average milking interval increased from 9.37 to 10.34 h, which was within the optimal range (9–10 h) for AMS operation. Milking stall occupancy and teat-cup attachment times gradually increased, whereas milking time showed no significant change. Somatic cell count initially increased but stabilized after three months. Milking efficiency ranged from 2.44 to 2.56 kg/min. In the system comparison, AMS-C showed shorter milking stall occupancy time, higher milking frequency, and higher milking efficiency than AMS-K, whereas AMS-K showed higher milk yield per milking, associated with longer milking interval. Across both systems, multiparous cows exhibited longer milking intervals and higher milk yields than primiparous cows in both AMSs. Theoretical milking capacity per AMS was 54.45 cows (primiparous) and 37.77 cows (multiparous) for AMS-K, sufficient for an average Korean dairy farm. Our results demonstrate that AMS-K achieved stable milking performance, milk quality, and operational efficiency comparable to the imported AMS. AMS-K shows great potential for practical applications and commercialization in Korean dairy farms.

## Simple Summary

Automatic milking systems (AMS) are increasingly being used worldwide to reduce labor demands, improve cow welfare, and enhance farm efficiency. However, their adoption in Korea remains limited, owing to high initial investment costs and a lack of technical validation for domestically developed models. In this study, we evaluated the milking performance of a newly developed domestic AMS (AMS-K) and compared it with that of a widely used commercial AMS (AMS-C) under identical management and housing conditions. The AMS-K was tested for three months using Holstein dairy cows to assess its milking efficiency, milk yield, and milk quality. Our results showed that the milking efficiency was comparable to that of imported AMSs, and somatic cell counts stabilized by the third month of operation of the system. Although AMS-K exhibited longer milking times than AMS-C, it demonstrated stable performance and a 100% success rate in automatic milking. The theoretical milking capacity of AMS-K was appropriate for the average herd size on Korean dairy farms. These results indicate that AMS-K is a technically reliable and field-applicable domestic AMS, supporting its potential commercialization and broader adoption in Korea’s dairy industry.

## 1. Introduction

Automatic milking systems (AMS), also known as a robotic milking system, were developed to automate the milking process, thereby reducing labor and improving cow welfare and farm efficiency [1]. Approximately 50,000 AMS units are installed worldwide, 90% of which are in Europe, 9% in Canada, and 1% in other countries [2]. In Korea, AMS usage has gradually expanded since its introduction in the Gyeonggi Province in 2006; however, as of 2024, only 3.3% of dairy farms have adopted AMS [3]. Nevertheless, owing to the aging farming population and shortage of successors, the adoption of AMS is expected to accelerate in the near future.

AMS improves labor efficiency by allowing cows to voluntarily enter the milking stall and be milked automatically, enhancing both animal welfare and quality of life for farmers, and providing positive effects such as increased milk yield through an optimized milking process [4,5]. However, the high initial investment cost required for AMS installation and barn modification remains a major hurdle to its wider adoption [6]. To address this issue, a domestically produced AMS that automates a series of processes, including concentrate feeding, teat washing, teat-cup attachment, milking and teat-cup removal, and enables cows to enter and exit the milking stall autonomously, has recently been developed in Korea [7].

With the growing adoption of AMS, researchers have actively investigated its effects on milk flow, milk quality, cow behavior, health, welfare, milking performance, and labor efficiency [5,8]. Among the various factors influencing AMS performance, automatic teat-cup attachment represents the most critical structural difference compared with conventional milking systems (parlors) [9]. Both consistency and efficiency of teat-cup attachment are essential for the successful operation of AMS farms, as attachment failure can decrease overall AMS efficiency, reduce milk yield, and increase the risk of mastitis [10]. In addition, the time required for teat-cup attachment may vary depending on udder conformation, teat position, and differences in teat cleaning and attachment mechanisms between AMS models [9]. Field-based evaluations of domestically developed AMS (AMS-K) are scarce, and it remains unclear whether an AMS-K can perform comparably to imported models under local farm conditions. Given the differences in herd size, barn design, and management practices in Korea, field-based validation of domestic AMS technology is needed.

The general goal of this research was to evaluate the performance of AMS-K under practical dairy farm conditions by scientifically assessing changes in milking characteristics and milk productivity during the initial operation. Furthermore, we compared the milking characteristics and efficiencies of primiparous and multiparous cows with those obtained using an imported commercial AMS (AMS-C). Practically, the study sought to provide evidence supporting the applicability and field readiness of domestic AMS technology for Korean dairy farms. Our results provide baseline information that supports the adoption and practical application of AMS-K in real farm environments.

## 2. Materials and Methods

### 2.1. Ethical Approval and Feeding Management

This study was conducted at the experimental dairy farm of the Dairy Science Division, National Institute of Animal Science (NIAS). All experimental procedures were approved by the Institutional Animal Care and Use Committee (IACUC) of NIAS (Approval No.: NAIS2022-0552).

The domestically produced AMS (AMS-K) and imported commercial AMS (AMS-C) were installed in open-type barns with identical layouts, each containing a feeding area and a loose-housing resting area bedded with sawdust. Barn temperature was regulated using mechanical fans according to the temperature–humidity index, and lighting was maintained through natural daylight supplemented with LED fixtures providing 150–200 lux. These environmental conditions ensured adequate welfare in the lying, feeding, and social areas throughout the study period. The cows were pre-trained according to the manufacturer’s manuals and equipped with transponder collars for individual identification. A total mixed ration (TMR) was provided twice daily (09:00 and 15:00) as the basal feed (Table 1), whereas the concentrate feed was automatically dispensed within the AMS based on each cow’s milk yield, days in milk (DIM), and parity.

The same group of cows was maintained throughout each experimental period, and no animals were removed or replaced at any stage of the study. All cows remained clinically healthy, and no cases of clinical mastitis were detected during the observation period.

### 2.2. Structure and Operation of Domestic Automatic Milking System (AMS-K)

**Figure 1:**
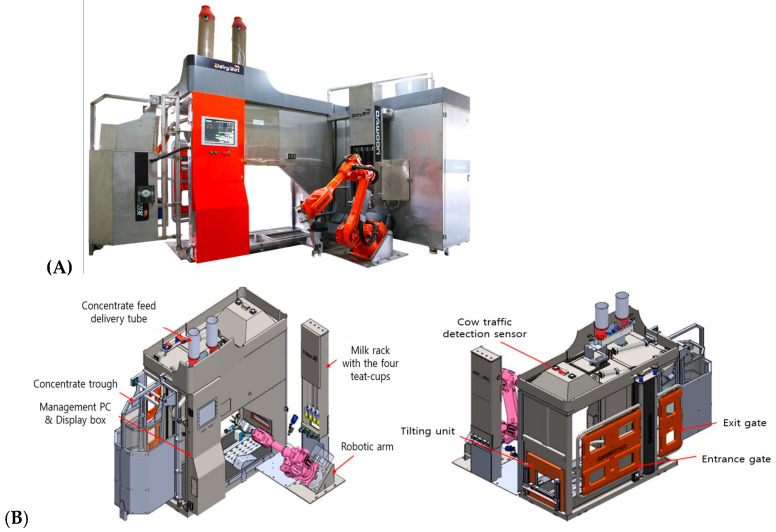
The domestic automatic milking system (AMS-K) used in this study. The system features a 6-axis robotic arm for independent teat-cup attachment and automated udder preparation. (**A**) Photograph of the AMS-K installed at the experimental dairy farm. (**B**) Schematic diagram illustrating the structural components and operational layout of the AMS-K.

AMS-K (Dairybot K1, Dawoon Industries, Incheon, Republic of Korea) consists of a single milking stall equipped with a concentrate feeder, a six-axis industrial robotic arm (HD Hyundai Robotics, Daegu, Republic of Korea), and four teat-cup 10s (Figure 1). The robotic arm detects teats using a 3D camera and sequentially attaches teat-cups via a gripper. Each teat-cup integrates the functions of teat cleaning, milking, and post-milking disinfection. The AMS-K was continuously operated for 3 months on 25 Holstein dairy cows (parity 1.98 ± 0.02, DIM 132.8 ± 58.2 days, milk yield 33.05 ± 12.6 kg/d), and milking characteristics were analyzed using data automatically collected in real time.

### 2.3. Operation of Domestic and Imported AMS According to Parity

For the comparative evaluation of milking characteristics between the two AMSs, a total of 50 Holstein cows were assigned to either AMS-K or AMS-C (Astronaut A4, Lely Industries, Maassluis, The Netherlands), and both groups were monitored over a three-month period. Cows were allocated to each AMS based on comparable baseline characteristics, including parity, days in milk (DIM), and average milk yield (AMS-K: parity 1.92 ± 1.16, DIM 136.38 ± 51.03 days, milk yield 33.20 ± 7.8 kg/d; AMS-C: parity 2.28 ± 1.28, DIM 137.01 ± 47.09 days, milk yield 34.39 ± 7.1 kg/d). Both AMSs had a single milking stall structure and were installed in front of a loose barn (15 m × 50 m per barn) using a free-flow movement system that allowed voluntary cow movement. AMS-C differs from AMS-K in terms of robotic arm control and teat-cleaning mechanism. Specifically, the AMS-C used a pneumatically controlled robotic arm, integrating a teat-cleaning brush, teat detector, and teat-cups [9]. All AMSs were automatically cleaned every six hours, with daily cleaning between 02:00 and 03:00 (Table 2). Cows that had not been milked for more than eight hours were manually guided to the milking machine once or twice daily by a caregiver. Teat-cup detachment at the end of the milking process was automatically controlled by milk flow rate.

### 2.4. Data Collection

All milking data were automatically recorded and collected in real-time using dedicated software for AMS (AMS-K, Dairybot view, Dawoon Industries, Incheon, Republic of Korea; AMS-C, Horizon, Lely Industries N.V., Maassluis, The Netherlands). The collected parameters included: (1) individual information (individual identification number, DIM, and parity), (2) milking performance indicators (milking stall occupancy time, milking time, teat-cup attachment time, milking frequency, and milking interval), and (3) milk production traits (per-milking and daily milk yield, milk flow rate, milk fat, milk protein, lactose, somatic cell count, mastitis index, and electrical conductivity).

The success of automated milking, milking stall occupancy time, and milking time were recorded by three trained observers who visually observed the milking process of each system for seven consecutive days (09:00–18:00). Successful milking was defined as a complete milking cycle without any human intervention. The number of teat-cup attachment attempts was calculated as four base attempts (one per teat) plus additional attempts. The milking stall occupancy time was defined as the total time from the entry of the cow to its exit from the milking stall. Milking time was defined as the occupancy time minus the teat-cup attachment time. The milking interval was defined as the time difference between consecutive milking of a cow. Milking frequency refers to the number of times a cow visits the milking stall for milking in a single day. Farms typically use milking capacity per AMS as an indicator to evaluate AMS efficiency [11]. The theoretical milking capacity per AMS was estimated as follows:

$$Milking\;capacity\left(cows\right)=\frac{Total\;milking\;hours\;per\;day}{Milking\;frequency\left(times/day\right)\times Milking\;time\left(h/milking\right)}$$

The total milking hours per day were set at 20 out of 24 h to account for routine automatic cleaning cycles, scheduled maintenance, and occasional technical downtime. This approach is aligned with previously published methods for estimating AMS throughput [11], which similarly adjusted operational time to reflect practical constraints in commercial dairy herds.

### 2.5. Statistical Analysis

All data were analyzed using SAS Enterprise Guide 7.1 (SAS Institute, Cary, NC, USA). Changes in milking characteristics of AMS-K over time were analyzed using a one-way ANOVA. The effects of AMS type and parity were analyzed using a general linear model (GLM). Statistical significance was set at *p* < 0.05, and Duncan’s multiple range test was performed for items where significance was confirmed to distinguish between means.

## 3. Results and Discussion

### 3.1. Milking Performance of AMS-K

The milking characteristics of AMS-K are summarized in Table 3. Milk yield per milking increased significantly from 13.81 kg (month 1) and 13.95 kg (month 2) to 15.99 kg (month 3) (*p* < 0.05), while daily milk yield increased by 4.01–7.52% in the third month (*p* < 0.05). Milk frequency decreased from 2.53 times/day (month 1) to 2.27 times/day (month 3) (*p* < 0.05), yet remained higher than that of conventional parlor milking (2 times/day). This pattern appears to be because voluntary visits by cows were active in the early stages of the AMS operation, after which the milking pattern gradually stabilized and was adjusted to a regular interval. The results of the present study are consistent with those of previous studies [12,13] that found that milk production increased as milking frequency increased. Melin et al. [13] reported that milk production increased by approximately 9% when milking frequency of AMS increased from 2.1 times/day to 3.2 times/day. However, the relationship between milking frequency and milk yield is not strictly linear but can be influenced by various factors such as the cow’s voluntary milking behavior, barn layout, and feed palatability [14]. Adjustments to milking frequency should consider both milking behavior and feeding management factors.

The milking interval increased from 9.37 h (month 1) to 10.34 h (month 3) during the operation period (*p* < 0.05). The results of this study correspond to an optimal milking interval of 9–10 h for AMS, as suggested by Mollenhorst et al. [15]. Excessively frequent milking (<6–8 h) can lead to decreased milk flow due to the stimulation of teat tissue and increased energy expenditure, whereas excessively delayed milking (>12 h) can lead to concerns about udder distension, increased somatic cell count, risk of mastitis, and decreased AMS efficiency [15].

Milking stall occupancy time and teat-cup attachment time significantly increased over the three-month period (*p* < 0.05), and the milking time was 8.55 to 8.69 min, with no significant difference depending on the operating period. These results appear to be longer than those of previous studies reporting the milking time of imported AMSs (6–7 min) [16,17]. Because excessive milking stall occupancy time, teat-cup attachment time, and milking time may affect both cow comfort and AMS efficiency [18,19], further research is needed to optimize these parameters.

The changes in milk composition, quality, and milk flow rate during the initial AMS-K operation are shown in Table 4. Somatic cell score tended to increase from the 1st month (5.06 units) to the 2nd month (5.19 units), but stabilized by the 3rd month (4.97 units). Van den Borne et al. [20] similarly reported a temporary increase in somatic cell count during the early adaptation period of AMS operation due to differences in teat cleaning and hygiene management compared to conventional milking.

The milking efficiency of the AMS-K ranged from 2.44 to 2.56 kg/min during 3 months of operation. The results of this study are similar to those of previous studies using imported AMSs (2.40–2.99 kg/min) [21,22]. Aerts et al. [21] noted that the highest performance of an AMS was achieved when the milking efficiency exceeded 2.40 kg/min, suggesting that the AMS-K is reliable in terms of technical performance.

### 3.2. Comparison of Milking Characteristics of AMS-K and AMS-C by the Parity of Dairy Cows

The comparison results of the milking performances of AMS-K and AMS-C are presented in Table 5. In both AMS-K and AMS-C, automatic milking was 100% successful for primiparous and multiparous cows, without any intervention by the manager. In addition, the number of attempts to attach the teat-cup did not differ by parity, but the AMS-K (primiparous 4.72 times, multiparous 5.01 times) showed attachment accuracy similar to that of AMS-C (primiparous 5.09 times, multiparous 5.04 times). The success rate of teat-cup attachment is directly related to the processing efficiency of the AMS and udder health [10,23], and maintaining high precision is essential for stable operation. An increase in the failure rate may result in stress in dairy cows, a decrease in the frequency of voluntary visits, an increase in milking time, and a decrease in milk production because of incomplete milking [5].

Milking stall occupancy and milking times were significantly longer in the AMS-K than in AMS-C (*p* < 0.05). Also, teat-cup attachment time of AMS-K was numerally longer than AMS-C. In AMS-K, the milking stall occupancy time and milking time increased by 3.43 and 2.01 min, respectively, in multiparous cows compared to primiparous cows (*p* < 0.05), whereas in AMS-C, the milking stall occupancy time (0.50 min) and milking time (0.14 min) increased in primiparous cows compared to multiparous cows (*p* < 0.05). In particular, the longer milking stall occupancy and milking time observed in AMS-K may be attributed to its sequential attachment process and the integrated teat-cleaning function within each teat-cup [9]. This multistep process increases the duration that the robotic arm remains positioned under the udder, slowing the initiation of milking compared with the AMS-C, which uses a pneumatically operated arm equipped with a dedicated cleaning brush. The increased arm movements and prolonged attachment time in AMS-K may induce restlessness in more sensitive cows, suggesting that further functional improvements are needed to enhance cow comfort.

Milking frequency was significantly higher in AMS-C (primiparous 2.74 times, multiparous 2.83 times) than in AMS-K (primiparous 2.35 times, multiparous 2.48 times) (*p* < 0.05) and higher in multiparous than primiparous cows across both systems (*p* < 0.05). Similarly to the present results, Rodriguez et al. [22] reported that milking frequency was higher in primiparous cows (2.49 times) than in multiparous cows (2.41 times), which was attributed to the lower milk flow rate and higher frequency of kick-offs during milking in primiparous cows than in multiparous cows.

The milking interval was longer in AMS-K (primiparous 10.04 h, multiparous 10.46 h) than in AMS-C (primiparous 8.01 h, multiparous 8.68 h) (*p* < 0.05), and was longer in multiparous cows (*p* < 0.05). Spolders et al. [24] explained that multiparous cows (6.95–10.28 h) had greater udder volume and storage capacity than primiparous cows (5.82–7.37 h), which delayed the increase in milk accumulation pressure and extended the milking interval. In contrast, Rodriguez et al. [22] found no difference between primiparous (9.54 h) and multiparous (9.56 h) cows. These variations may arise from differences in milk yield, intra-udder pressure, voluntary visiting behavior, and feed palatability [15,24]. Furthermore, the higher milk yield and more advanced mammary development typically observed in multiparous cows may increase their capacity for milk accumulation, contributing to longer intervals between milkings. Therefore, the milking interval should be adjusted by considering parity-related physiological differences and individual cow characteristics to optimize both AMS efficiency and udder health.

Both AMS-K and AMS-C showed significantly higher milk yields per milking and per day in multiparous cows than in primiparous cows (*p* < 0.05). Milk yield per milking was higher in AMS-K (primiparous 13.61 kg, multiparous 17.86 kg) compared with AMS-C (primiparous 11.58 kg, multiparous 13.37 kg) (*p* < 0.05). This difference likely resulted from the longer milking interval.

Milking efficiency was greater in AMS-C (2.80 kg/min) than with AMS-K (2.24 kg/min) (*p* < 0.05). Rodriguez et al. [22] reported that multiparous cows have higher milk yields and flow rates, resulting in a higher milking efficiency (2.03 kg/min), whereas primiparous cows have a milking efficiency of 1.91 kg/min. In comparison, the results of the present study showed no difference in milking efficiency according to parity of dairy cows; however, AMS-K and AMS-C showed higher milking efficiency.

The theoretical milking capacity per AMS was lower for AMS-K (primiparous 54.45 cows, multiparous 37.77 cows) than for AMS-C (primiparous 63.66 cows, multiparous 66.45 cows). Considering the number of lactating dairy cows per domestic farm (35.85 cows) [25], a single AMS-K would currently be sufficient for continuous milking operations on domestic farms. However, as herd size increases in the future with farm intensification, milking throughput may become a limiting factor. Therefore, to support future herd expansion and avoid performance bottlenecks, improving the milking capacity of AMS-K should be considered.

Recent work indicates that the productivity and milk quality responses observed when transitioning from conventional milking to AMS can vary substantially depending on farm environment, housing structure, management practices, and cow-level characteristics [26]. Moreover, several recent reviews highlight the limited availability of empirical AMS data from Asian countries, including Korea, and emphasize the need for region-specific validation [27]. In this context, evaluating the performance of a domestically developed AMS under practical field conditions is essential for determining its suitability for Korean dairy farms. The present study provides such evidence by directly comparing AMS-K with a commercial AMS under standardized management conditions, thereby supporting its technical reliability and potential for broader on-farm adoption.

This study has several limitations. First, the experiment was conducted on a single commercial farm with a limited number of cows, which restricts the generalizability of the findings. In addition, the observation period focused primarily on the early adaptation phase, limiting our ability to draw conclusions about long-term performance and health outcomes. Economic evaluations and maintenance requirements of the two AMS models were also not assessed in this study. Future research should include multi-farm trials, long-term monitoring, and comprehensive economic analyses to better determine the practical value and scalability of AMS-K in Korean dairy production systems.

## 4. Conclusions

Operating the domestic AMS-K for three months increased milk yield, stabilized milk quality, and milking efficiency, and achieved a level of performance comparable to that of imported AMSs. Although AMS-K required slightly longer operation times owing to its integrated milking structure, it maintained stable efficiency and hygiene. Based on its theoretical capacity, a single AMS-K can efficiently serve an average-sized Korean dairy herd. Therefore, AMS-K represents a reliable domestic technology with a strong potential for practical application and commercialization in Korean dairy farming.

## Figures and Tables

**Table 1:** Ingredient and chemical composition of the total mixed ration (TMR) and concentrates fed to Holstein dairy cows.

Item	TMR (% of DM)	Concentrations (% of DM)
Ingredient composition		
Corn silage	18.01	-
Pasture hay	26.34	-
Alfalfa	8.08	-
Timothy	8.27	-
Soybean meal	2.01	-
Beet pulp	6.02	-
Cottonseed	8.06	-
Commercial concentrate	21.86	-
Calcium carbonate	0.07	-
Vitamin-mineral mix	0.07	-
Chemical analysis		
Moisture (% of *as fed*)	31.66	12.27
Crude protein	14.97	22.49
Ether extract	3.83	4.33
Crude fiber	25.81	9.18
Crude ash	8.52	7.06
Neutral detergent fiber	54.24	32.44
Acid detergent fiber	32.24	15.96
TDN ^1^	65.70	83.97
DE ^2^, Mcal/kg	2.89	3.70
NEL ^3^, Mcal/kg	1.56	1.93

^1^ TDN: Total digestible nutrients; ^2^ DE: Digestible energy; ^3^ NE_L_: Net energy of lactation.

**Table 2:** Technical specifications of domestic and commercial automatic milking systems.

Specifications	AMS-K ^1^	AMS-C ^2^
Milking facility design	Single stall	Single stall
Robot arm	6-axis industrial manipulator	Special manipulator
Udder cleaning	Washing within teat-cup	Cleaning brush
Time limit for milking, h	6 h after a previous milking	6 h after a previous milking
Milking delay alert, h	12 h after a previous milking	12 h after a previous milking

^1^ AMS-K is a domestic automatic milking system. ^2^ AMS-C is a commercial automatic milking system.

**Table 3:** Changes in milking performance during the operation period of domestic automatic milking system (AMS-K).

Item	Operation Period (Month)			SEM	*p*-Value
	1	2	3		
Milk yield (kg/milking)	13.81 ^b^	13.95 ^b^	15.99 ^a^	0.08	<0.001
Daily milk yield (kg)	34.90 ^b^	33.76 ^c^	36.30 ^a^	0.24	<0.001
Milking frequency (no.)	2.53 ^a^	2.42 ^b^	2.27 ^c^	0.01	<0.001
Milking intervals (hr)	9.37 ^c^	9.68 ^b^	10.34 ^a^	0.04	<0.001
Milking-stall occupation (min)	10.35 ^c^	10.82 ^b^	11.39 ^a^	0.07	<0.001
Teat-cup attachment (min)	1.80 ^c^	2.13 ^b^	2.82 ^a^	0.03	<0.001
Milking time (min)	8.55	8.56	8.69	0.07	0.724

^a,b,c^ Mean in the same row followed by different letters are significant (*p* < 0.05).

**Table 4:** Changes in milk composition and traits during the operation period of domestic automatic milking system (AMS-K).

Item	Operation Period (Month)			SEM	*p*-Value
	1	2	3		
Milk fat (%)	3.47	3.53	3.52	0.02	0.083
Milk protein (%)	3.38 ^b^	3.41 ^a^	3.31 ^c^	0.01	0.003
Lactose (%)	4.74 ^a^	4.73 ^a^	4.69 ^b^	0.01	<0.001
Somatic cell score ^1^ (units)	5.31 ^b^	5.45 ^a^	5.30 ^c^	0.01	<0.001
Mastitis detection index	6.59 ^b^	6.45 ^b^	7.04 ^a^	0.04	<0.001
Milk electrical conductivity (mS/cm)	5.37 ^a^	5.35 ^a^	5.26 ^b^	0.01	<0.001
Maximum milk conductivity (mS/cm)	5.88 ^a^	5.92 ^a^	5.75 ^b^	0.01	<0.001
Blood in milk (mg/L)	0.43	0.20	0.11	0.08	0.365
Milk flow (kg/min)	0.60 ^c^	0.62 ^b^	0.69 ^a^	0.00	<0.001
Maximum milk flow (kg/min)	4.77 ^b^	4.80 ^b^	5.12 ^a^	0.02	<0.001
Milking efficiency (kg/min)	2.45 ^b^	2.44 ^b^	2.56 ^a^	0.01	<0.001

^a,b,c^ Mean in the same row followed by different letters are significant (*p* < 0.05). ^1^ Somatic cell score calculated as log_10_ (somatic cell count/mL).

**Table 5:** Comparison of milking performance by parity in cows milked with domestic and commercial automatic milking systems.

Item	AMS-K ^1^		AMS-C ^2^		SEM	*p*-Value		
	Primiparous	Multiparous	Primiparous	Multiparous		Parity ^3^ (P)	AMS (A)	P × A
Cows per AMS (no.)	15	10	12	13	-	-	-	-
Days in milking (day)	186.72	185.88	182.75	190.95	4.13	0.658	0.948	0.586
Milking success (%)	100	100	100	100	-	-	-	-
Teat-cup attachment frequency (times/milking)	4.72	5.01	5.09	5.04	0.79	0.411	0.177	0.249
Milking-stall occupation (min)	9.38	12.81	6.88	6.38	0.56	0.248	<0.001	0.036
Teat-cup attachment (min)	2.26	2.42	0.94	0.90	0.07	0.411	0.177	0.249
Milking time (min)	7.21	9.22	5.22	5.08	0.17	0.001	<0.001	<0.001
Milking frequency (times/day)	2.35	2.48	2.74	2.83	0.03	0.321	<0.001	0.107
Milking interval (hr)	10.04	10.46	8.01	8.68	0.13	0.028	<0.001	0.617
Milk yield per milking (kg)	13.61	17.86	11.58	13.37	0.25	<0.001	<0.001	0.006
Milk yield per day (kg)	32.33	40.16	32.93	39.25	0.54	<0.001	0.870	0.426
Milking efficiency (kg/min)	2.20	2.28	2.73	2.87	0.06	0.325	<0.001	0.764
Milk flow rate (kg/min)	0.56	0.89	0.95	0.93	0.01	<0.001	<0.001	<0.001
Milk fat (%)	3.31	3.46	3.95	4.05	0.05	0.141	<0.001	0.764
Milk protein (%)	3.42	3.22	3.20	3.19	0.02	0.004	0.001	0.007
Lactose (%)	4.89	4.57	4.72	4.63	0.02	<0.001	0.088	<0.001
Somatic cell score ^4^ (units)	5.37	5.32	4.98	5.21	0.01	<0.001	<0.001	0.002
Milking capacity ^5^ (cows/unit)	54.45	37.77	63.66	66.45	-	-	-	-

^1^ AMS-K is a domestic automatic milking system. ^2^ AMS-C is a commercial automatic milking system. ^3^ Parity: Primiparous cows in 1st lactation and multiparous cows in ≥2nd lactation. ^4^ Somatic cell score calculated as log_10_ (somatic cell count/mL). ^5^ Milking capacity refers to the theoretical number of cows that can be milked by a single AMS unit within a 24 h period.

## Data Availability

The data presented in this study are available on request from the corresponding author upon reasonable request. Access to the dataset is restricted by the institutional data-sharing policy of the National Institute of Animal Science.
